# Thrombolytic treatment in stroke mimic, inevitable but fortunately safe: An observational study from Iran

**Published:** 2019-10-07

**Authors:** Sara Esmaeili, Motahareh Afrakhteh, Maryam Bahadori, Seyedeh Fahimeh Shojaei, Rezan Ashayeri, Masoud Mehrpour

**Affiliations:** 1 Department of Neurology, Student Research Committee, Firoozgar Hospital, Iran University of Medical Sciences, Tehran, Iran; 2 Firoozgar Clinical Research and Development Center, Iran University of Medical Sciences, Tehran, Iran; 3 Department of Neurology, Firoozgar Hospital, Iran University of Medical Sciences, Tehran, Iran

**Keywords:** Thrombolytic Therapy, Stroke, Iran

## Abstract

**Background:** A number of patients with symptoms of acute cerebral ischemia may have other causes called stroke mimics (SM). The prevalence of SM can be as high as 31% in some reports, and these patients are potentially at the risk of intravenous thrombolysis (IVT) therapy and its complications. This study was designed to determine the prevalence of our center’s SM (Firoozgar Hospital) among patients who received IVT, their baseline characteristics, final diagnoses, and outcomes.

**Methods:** We reviewed the medical records of all patients who received IVT between June 2015 and May 2018. The following variables were collected: demographic characteristics, past medical history, onset-to-needle (OTN) time, door-to-needle (DTN) time, National Institutes of Health Stroke Scale (NIHSS) score at admission, brain imaging, and all paraclinic findings. Functional outcome at discharge based on modified Rankin Scale (mRS) was also assessed.

**Results:** 10 out of 165 (6.0%) patients including 8 men and 4 women were finally diagnosed with SM. The median age and NIHSS score at presentation were 60 years and 7, respectively. Final diagnoses were seizure (n = 6), hemiplegic migraine (n = 2), conversion (n = 1), and alcohol intoxication (n = 1). All patients were discharged with a mRS score of 0 and 1 without experiencing any thrombolytic adverse effects.

**Conclusion:** None of the patients with SM experienced any adverse effect of tissue plasminogen activator (tPA) including hemorrhage and all of them reached good mRS score. This shows that tPA is generally safe and the risk of treating patients with SM is very low and making a vital treatment decision may outweigh the risk of neglected cases in a time-sensitive setting.

## Introduction

Stroke mimics (SM) cases are patients with symptoms of focal neurologic deficit simulating acute ischemic stroke (AIS) symptoms who are diagnosed as stroke patients but there is no tissue infarction. The prevalence of SM among those with the initial diagnosis of AIS on admission has been reported between 4.8% and 31.0% in different reports.^1^

Diagnosis of all patients with SM may be challenging in the emergency situation. Hence, these patients may receive intravenous recombinant tissue plasminogen activator (rtPA) therapy if they arrive in time.^2^ The most important adverse effect of intravenous thrombolysis (IVT) treatment is intracranial hemorrhage (ICH). It is important to be aware of rtPA safety and clinical outcomes in SM patients who were treated with IVT.^1^^-^^3^

## Materials and Methods

We reviewed the medical records of all patients who received rtPA at our stroke center in Firoozgar Hospital, affiliated to Iran University of Medical Sciences, Tehran, Iran, between June 2015 and May 2018. Clinical documents were evaluated in these patients to determine SM cases. All patients underwent brain computed tomography (CT) scan before and 24 hours after receiving IVT. Finding no infarction on control CT scan, magnetic resonance imaging (MRI) was also performed. Normal MRI was not the only criterion to consider a patient as an SM case, but the exact diagnosis was established in all SM patients based on vague symptoms and neurologic syndromes lacking in defined localization. All SM cases received 0.9 mg/kg of Alteplase. The final diagnosis of SM was based on normal brain MRI combined with clinical diagnosis of other diagnostic and paraclinical laboratory data including electroencephalogram (EEG) and blood samples together with the corresponding specialist consultations. The following variables were collected: demographic characteristics, National Institutes of Health Stroke Scale (NIHSS) score at admission, past medical history, onset-to-needle (OTN) time, door-to-needle (DTN) time, dose of rtPA, findings of brain imaging, laboratory results which led to a final diagnosis other than stroke, and modified Rankin Scale (mRS) at discharge. All collected data were reviewed by a vascular neurologist.

## Results

Among a total of 165 patients who received IVT during this period, 10 patients with SM were identified (6%) without evidence of acute ischemic lesion on MRI, following with a final diagnosis other than stroke (Table 1). There were eight men and four women, the median age was 60 years (range: 31-87). The median NIHSS score on admission was 7 (range: 3-19). The most common SM syndrome was seizure (n = 6) followed by hemiplegic migraine (n = 2), one with conversion reaction, and one patient was discharged with the final diagnosis of alcohol intoxication. One of the patients was later found to be pregnant at the time of stroke.

Six patients had risk factors for stroke and one patient had the history of previous ischemic stroke.

None of these cases experienced ICH or other rtPA complications. All patients were discharged home with favorable functional outcome defined by mRS ≤ 1 (Table 1).

## Discussion

Administration of intravenous (IV) rtPA is more efficient when it occurs within the first 90 minutes after symptom onset. It is important to make DTN time as short as possible in emergency setting. This effort may lead to rtPA administration in patients who present with symptoms mimicking AIS.^1^^-^^5^ When patients are presented at emergency room with symptoms equal to acute stroke, there has always been debate whether it is harmful to use rtPA and it is rational to waste the critical time in order to reach a final correct diagnosis or to risk using IV rtPA as soon as possible in order not to lose the vital time.^4^ This study shows that 10 patients with SM were finally detected among 165 patients in the aforementioned time period who received rtPA. These patients received rtPA due to their similar symptoms to stroke in critical time. None of them experienced any adverse effects including ICH and all were discharged with favorable outcome (mRS ≤ 1) without any further complications. These results confirm the generally safe side view of IV rtPA and the necessity of rapidly deciding to use tPA in emergency department for patients without known contraindications who are admitted with symptoms similar to stroke in critical time in order not to lose vital time for its efficiency. These results are in favor of previous studies which found IV rtPA safe.^1^^-^^5^

**Table 1:** Patients data in the report

**Case**	**Age ** **(year)/sex**	**Clinical syndrome**	**Previous ** **stroke ** **or TIA**	**Brain CT ** **on ** **admission**	**Follow up ** **brain CT ** **and MRI**	**DTN ** **(minute)**	**OTN ** **(minute)**	**Stroke risk ** **factors**	**Initial ** **NIHSS**	**Final ** **diagnosis**	**rtPA ** **complication**	**mRS ≤ 1 at ** **discharge**
1	39/F	Dysarthria, QP	No	Normal	Normal	50	255	No	5	Seizure	No	Yes
2	87/M	GA	Yes (stroke)	Old infarction	Old infarction	90	170	AF, HTN	3	Seizure	No	Yes
3	65/M	GA, disorientation	No	SVD	SVD	180	220	No	13	Seizure	No	Yes
4	53/M	HP, confusion	No	SVD	SVD	40	100	CAD	7	Seizure	No	Yes
5	76/F	HP	No	SVD	SVD	30	85	DM, HTN	6	seizure	No	Yes
6	55/F	Broca’s aphasia, HP	No	Normal	Normal	25	145	CAD, HTN, HLP	12	Migraine	No	Yes
7	31/M	HP, hemihypesthesia, headache	No	Normal	Normal	55	135	HLP	5	Migraine	No	Yes
8	60/M	Dysarthria, HP	No	SVD	SVD	45	90	No	4	Alcohol intoxication	No	Yes
9	55/M	Loss of consciousness, dysarthria, ataxia	No	Old infarction	Normal	40	80	HTN	4	Seizure	No	Yes
10	42/F	Right HP	No	Normal	Normal	35	90	No	4	Conversion disorder	No	Yes

QP: Quadriparesis; HP: Hemiparesis; GA: Global aphasia; TIA: Transient ischemic attack; SVD: Small vessel disease; DTN: Door-to-needle; OTN: Onset-to-needle; AF: Atrial fibrillation; HTN: Hypertension; CAD: Coronary artery disease; HLP: Hyperlipidemia; NIHSS: National institutes of health stroke scale; CT: Computed tomography; MRI: Magnetic resonance imaging; DM: Diabetes mellitus; rtPA: Recombinant tissue plasminogen activator; mRS: Modified Rankin scale

Moreover, in a meta-analysis, the prevalence of symptomatic ICH among patients with SM who were treated with IVT was ≤ 0.5%.^1^ The prevalence of SM is different among various studies.

In one study on 295 patients with SM, brain tumors were the top list of final diagnosis (10.5%) followed by seizure and sepsis, respectively.^5^ In some other studies seizure, conversion disorders, and migraine were more prevalent.^1^^-^^7^ In this study, seizure was responsible for 6 (60%) SM cases, followed by hemiplegic migraine.

Patients with history of stroke may present to emergency room with toxic, metabolic, infectious, or other causes which mimic stroke. Fever might be a clue in favor of SM diagnosis, but the presence of stroke risk factors in one patient and the history of transient ischemic attack (TIA) in another one was convincing reasons in order not to miss an acute treatment. Alcohol intoxication was the cause of one SM in the present study.^5^^,^^7^

Regarding patients’ symptoms, it has been suggested that the absence of facial paresis and NIHSS score ≤ 5 are associated with SM diagnosis.^3^^,^^4^ In our study, only one patient had experienced facial weakness associated with severe headache whose final diagnosis was hemiplegic migraine. Lack of facial paresis in our patients could have been a valuable predictor for SM. Some other studies also emphasized on a low NIHSS score role as a predictor factor for SM.^4^

In our study, the NIHSS score was more than 5 in 5 patients. Number of patients with SM in our study was only 10, which was too small to determine the importance of gender effect or other underlying factors as well as the presence of known stroke risk factors and a low NIHSS score as predictors for SM diagnosis. Nevertheless, there was a disagreement between our study and some previous studies considering mentioned baseline characteristics. Further studies on more patients regardless of receiving rtPA therapy may show the predictive strength of these factors.

## Conclusion

Our research indicates the safe profile of IV rtPA in SM cases in our center which matches the results of previous studies. Although IVT potentially has adverse effects, the benefit of administrating IVT in its maximum efficient time for patients admitted to the emergency room with symptoms indicating AIS probably outweighs the complication risks even if the patient final diagnosis finishes with SM.

## Notes:

**How to cite this article:** Esmaeili S, Afrakhteh M, Bahadori M, Shojaei SF, Ashayeri R, Mehrpour M. Thrombolytic treatment in stroke mimic, inevitable but fortunately safe: An observational study from Iran. Iran J Neurol 2019; 18(4): 172-5.
